# Fabrication of Biocompatible Electrospun Poly(ε-caprolactone)/Gelatin Nanofibers Loaded with *Pinus radiata* Bark Extracts for Wound Healing Applications

**DOI:** 10.3390/polym14122331

**Published:** 2022-06-09

**Authors:** Jessica Borges-Vilches, Irem Unalan, Katherina Fernández, Aldo R. Boccaccini

**Affiliations:** 1 Laboratory of Biomaterials, Department of Chemical Engineering, Faculty of Engineering, Universidad de Concepción, Concepción 4030000, Chile; jessborges@udec.cl (J.B.-V.); kfernandeze@udec.cl (K.F.); 2 Institute of Biomaterials, Department of Materials Science and Engineering, Friedrich-Alexander-University Erlangen-Nuremberg, Cauerstraße 6, 91058 Erlangen, Germany; irem.unalan@fau.de

**Keywords:** poly(ε-caprolactone), gelatin, *Pinus radiata* bark extracts, electrospun nanofibers, wound healing

## Abstract

In this study, poly(ε-caprolactone) (PCL)/gelatin (GEL) electrospun nanofibers loaded with two different concentrations of *Pinus radiata* bark extracts (PEs) were fabricated via electrospinning for wound healing applications. The effects of incorporating PE into PCL/GEL electrospun nanofibers were investigated regarding their physicochemical properties and in vitro biocompatibility. All electrospun nanofibers showed smooth, uniform, and bead-free surfaces. Their functional groups were detected by ATR-FTIR spectroscopy, and their total phenol content was measured by a Folin–Ciocalteu assay. With PE addition, the electrospun nanofibers exhibited an increase in their wettability and degradation rates over time and a decrease in their tensile stress values from 20 ± 4 to 8 ± 2 MPa for PCL/GEL and PCL/GEL/0.36%PE samples, respectively. PE was also released from the fibrous mats in a rather controlled fashion. The PCL/GEL/0.18%PE and PCL/GEL/0.36%PE electrospun nanofibers inhibited bacterial activity at around 6 ± 0.1% and 23 ± 0.3% against *E. coli* and 14 ± 0.1% and 18 ± 0.2% against *S. aureus* after 24 h incubation, respectively. In vitro cell studies showed that PE-loaded electrospun nanofibers enhanced HaCaT cell growth, attachment, and proliferation, favoring cell migration towards the scratch area in the wound healing assay and allowing a complete wound closure after 72 h treatment. These findings suggested that PE-loaded electrospun nanofibers are promising materials for antibiotic-free dressings for wound healing applications.

## 1. Introduction

Wound healing is a dynamic and complex process involving hemostasis, inflammation, proliferation, and remodeling stages, as well as engaging a range of cells, genes, and cytokines for repairing the injured tissue [1,2]. Natural and synthetic traditional materials such as bandages, gauzes, and cotton wool have been used for protecting wounds and absorbing exudates [2]. However, they have no therapeutic effects on the different wound healing stages. Therefore, many efforts have been dedicated to developing new wound dressings with beneficial properties for wound healing [3,4]. Materials such as gelatin (GEL) [5,6], chitosan [2,4,7], alginate [8], collagen [9], polycaprolactone (PCL) [5,10,11], poly(α-L-glutamic acid) [12], antibiotics [13,14], and phytotherapeutics [5,10,11,15,16] are being used for such purpose. 

GEL is considered a promising biomaterial due to its biocompatibility and biodegradability, amphoteric character, good affinity with proteins, and capacity for potential modifications [17]. GEL promotes cell adhesion and proliferation when it is used in wound dressing applications [5,18]. On the other hand, PCL is a synthetic aliphatic polyester widely used in biomedicine, food, and other industrial applications [10,11,19] due to its biocompatibility, biodegradability, and desired mechanical properties [20]. However, certain disadvantages limit the use of both polymers as wound dressings. For instance, GEL exhibits poor mechanical strength [20,21], whereas PCL has a low degradation rate in aqueous solution and its hydrophobic nature limits cell adhesion [10,19]. Thus, PCL/GEL blends with improved mechanical and biological features have been developed to overcome those drawbacks [5,20,21,22]. 

Plant-derived metabolites have been widely used by humans for hundreds of years due to their healing properties [10,23]. Among them, the *Pinus radiata* bark extracts (PEs), composed of a high concentration of condensed tannins [24], have shown suitable antioxidant and anti-inflammatory properties, among other bioactivities [25,26]. However, the low solubility, high sensitivity against environmental changes, instability in physiological medium, and volatility of phenolic compounds have limited their use in biomedicine [10,15]. Electrospinning of PE–biopolymer systems could provide protection to PE against external factors to which the bark extract is vulnerable (light, heat, moisture, and oxidation). Electrospinning has been established as an outstanding and versatile technique to produce electrospun fibers with high specific surface area, excellent fluid drainage, and controlled drug delivery [5,10,11,19]. In the literature, different bioactive molecules have been entrapped into PCL/GEL electrospun fibers for wound dressing applications. For instance, Ramalingam et al. [20] demonstrated that natural herbal extracts (*Gymnema sylvestre*) loaded into PCL/GEL electrospun nanofibrous structures improved the antimicrobial activity of their materials. According to Mohamadi et al. [21], PCL/GEL nanofibers containing 20% *w/w* coconut oil improved biocompatibility and antibacterial activity against *S. aureus* and *E. coli* bacteria, which are desirable for wound healing. Similarly, Unalan et al. [5] fabricated PCL/GEL electrospun nanofibers loaded with clove essential oil (CLV). Their results showed that PCL/GEL/CLV nanofibers exhibited excellent antibacterial activity against *S. aureus* and *E. coli* bacteria and were considered promising candidates for wound healing applications.

According to the authors’ knowledge, incorporating PE into nanofibrous structures of PCL/GEL for biomedical applications has not been investigated before. Thus, this study aimed to develop PCL/GEL electrospun nanofibers loaded with *Pinus radiata* bark extracts for wound healing applications for the first time. In this regard, the effects of loading different PE concentrations (0.18% and 0.36% *w/w*) into PCL/GEL electrospun nanofibers were analyzed in terms of surface morphology, average fiber diameter, functional groups, total phenol content, wettability, degradation and release behavior, antibacterial activity, cell biocompatibility, and in vitro wound healing. 

## 2. Materials and Methods

### 2.1. Materials

Gelatin (type A, 300 g Bloom), glacial acetic acid (GAA, used as a solvent), PCL (Mw = 80,000 Da), fetal bovine serum (FBS, F2442), Folin–Ciocalteu reagent (F9252), and sodium carbonate were purchased from Sigma-Aldrich (Darmstadt, Germany). Phosphate-buffered saline (PBS, biotech grade, pH = 7.4) was obtained from Merck. The microorganism strains of *S. aureus* (ATCC25823) and *E. coli* (ACTC25922) were used. Luria/Miller agar (X969.1) and lysogeny broth medium (Luria/Miller, 6673.1) were supplied by Carl Roth GmbH (Karlsruhe, Germany). A human keratinocyte (HaCaT) cell line was obtained from Cell Lines Services GmbH (CLS, 300493, Baden-Württemberg, Germany). Dulbecco’s modified Eagle’s medium (DMEM, 31885-023), trypsin/EDTA (25200-056), and penicillin/streptomycin (PS, 15140-122) were purchased from Thermo Scientific (Schwerte, Germany). *Pinus radiata* bark was supplied by Technological Development Unit (UDT, Concepción, Chile). All reagents and solvents were of analytical grade. 

### 2.2. Pinus radiata Bark Extract Production 

*Pinus radiata* bark extracts were produced through a pilot-scale extraction process, as described by Bocalandro et al. [24] For this purpose, a reactor volume of 4 m^3^ and a vapor heating system composed of a shell and a tube heat exchanger with 6 m^2^ heat transference area were used. In addition, a recirculation circuit for the extracted solution was implemented. Briefly, the *Pinus radiata* bark was ground with a double-knife mill to an average size lower than 20 mm. Then, the bark was dried at room temperature until a humidity of 24.5% (*dry weight*), and 100 kg (*dry weight*) of bark was soaked in an ethanol/water solution at a 1:20 ratio (*w/v*) for 120 min at 120 °C. Subsequently, the ethanol was evaporated under a vacuum (absolute pressure 0.05 bar) at room temperature. Thus, the water-insoluble particulate material after decanting and the water-soluble polyphenol fraction were obtained. Finally, the water-soluble polyphenols were lyophilized at room temperature and the obtained extracts were stored in sealed amber glass containers for further analysis.

### 2.3. Preparation of Electrospinning Solutions

The electrospinning solutions were fabricated according to the protocol established by Unalan et al. [5], with slight modifications. Briefly, GEL powder (4.8% *w/w*) was dissolved in 10 mL of GAA (90% *v/v*) at 45 °C for 4 h. PCL pellets (1.12 g) were added to the previous solution and stirred overnight at room temperature. After 30 min of adding PCL pellets to prepare the PE-containing solution, different concentrations of PE (0.18% and 0.36% *w/w*) were separately added to each PCL/GEL solution. All prepared solutions were stirred overnight at room temperature to achieve homogeneity. Finally, each solution was sonicated for 15 min at room temperature before electrospinning.

### 2.4. Electrospinning Process 

The electrospinning process was carried out as previously described by Unalan et al. [5]. Briefly, PCL/GEL and PE-loaded PCL/GEL solutions were separately loaded into a plastic syringe (3 mL) fitted with a 21G needle. A commercially available device (Electrospinning Starter Kit, Linari Engineering Srl; Pisa; Italy) was used to produce the electrospun nanofibers by applying a voltage of 18 kV. The distance between the aluminum foil-wrapped collector and the needle tip was 12 cm. The flow rate used to produce electrospun nanofibers was 0.6 mL h^−1^. Finally, electrospinning was carried out under defined environmental conditions (temperature: 23 ± 2 °C and relative humidity: 26 ± 2%). The electrospun nanofibers were stored at 4 °C in the dark until further analysis. The nomenclature used to identify the electrospun nanofibers was PCL/GEL, PCL/GEL/0.18%PE, and PCL/GEL/0.36%PE. The above percentages (*w*/*w*) indicate the PE content loaded into each PCL/GEL solution.

### 2.5. Characterization of Electrospun Nanofibers 

The surface morphology of the electrospun nanofibers was analyzed by scanning electron microscopy (SEM) (ETH: 2 kV, Everhart-Thornley detector (SE2), AURIGA base 55, Carl Zeiss). The samples were coated with a layer of gold before SEM analysis. The average fiber diameter was calculated using 50 random pores obtained from the SEM images in ImageJ software (NIH, Bethesda, MD, USA).

The functional groups of PE, PCL/GEL, and PE-loaded PCL/GEL electrospun nanofibers were identified by attenuated total reflection Fourier-transform infrared (ATR-FTIR) spectroscopy (RA_nity-1S, Shimadzu, Duisburg, Germany). The spectra were measured in a wavenumber range between 4000 and 400 cm^−1^ using a spectral resolution of 4 cm^−1^ and 32 scans. 

The total phenol content (TPC) of the PE-loaded PCL/GEL electrospun nanofibers was determined by a Folin–Ciocalteu assay [27] with modifications. In this assay, 2 mL of methanol was added to 10 mg of nanofiber for 24 h. After that time, 2 mL of Folin–Ciocalteu reagent (diluted in deionized water in a ratio of 1:9) and 4 mL of sodium carbonate (7.5% *wt*.) were added to the above solution. The mixture was stored for 1.5 h in a dark room. Then, the absorbance of each sample and the blank (deionized water) was measured at 765 nm in a UV-Vis spectrophotometer (Specord 40, Analytik Jena GmbH, Jena, Germany). The measurements were performed in triplicate and the results were expressed in units of milligrams of equivalent gallic acid per milligram of fiber. 

The wettability of the electrospun nanofibers was analyzed by contact angle measurements using a sessile drop method in a contact angle meter (Drop Shape Analyzer, DSA 30, CA Measurement setup, Kruess GmbH, Hamburg, Germany). The electrospun nanofibers were placed on a glass slide before testing, and a drop of deionized water was dropped onto their surface. Three measurements were performed at different points on the surface of the same electrospun nanofiber, and the average value was determined. 

#### 2.5.1. Mechanical Properties of Electrospun Nanofibers

A uniaxial tensile test was performed with the electrospun nanofibers to evaluate their mechanical properties using a universal testing machine Instron 5967 (Instron GmbH, Darmstadt, Germany). In this test, the electrospun nanofibers were cut in a rectangular shape (0.5 cm width and 4 cm length) and fixed in a suitable paper square framework. The measurements were performed at a 1 mm/min crosshead speed using a 100 N load cell. Mechanical properties such as Young’s modulus, tensile strength, and elongation at break were calculated from the tensile stress–strain curves. These measurements were performed six times for each electrospun nanofiber, and an average value was reported.

#### 2.5.2. In Vitro Degradation Test

An in vitro degradation test was performed with the electrospun nanofibers as described previously [10]. Briefly, each electrospun nanofiber was cut into 3 × 3 cm^2^ pieces and immersed in 10 mL of PBS (1×, pH = 7.4). Then, the samples were incubated at 37 °C and 110 rpm for 1, 3, 7, 14, 21, 28, and 35 days. After each time interval, the samples were taken out from the PBS, rinsed with Milli-Q water, and dried at 37 °C for 3 days before the measurement. The weight loss of each electrospun nanofiber was calculated according to Equation (1):

(1) $$Weight\;loss\;\left(\%\right)=\frac{W_{initial}-W_{dry}}{W_{initial}}*100$$

where *W_initial_* and *W_dry_* are the initial and dry weight of fibers, respectively. This test was performed in triplicate for each sample. In addition, the chemical bonds of the electrospun nanofibers after drying were analyzed by ATR-FTIR spectroscopy.

#### 2.5.3. Determination of PE Composition

The polyphenol composition of PE was identified and quantified using a reversed-phase high-performance chromatography (RP-HPLC)–mass spectrometry (MS) system coupled with a diode array detector (DAD). This analysis was performed following the protocol proposed by Bocalandro et al. [24]. Briefly, 10 µL of the sample was filtered with Phenex-RC 15 mm syringe filters. Then 0.2 µm was injected three times (Phenomenex, Torrance, USA) using a mobile phase composed of 1% acetic acid (phase A) and acetonitrile (phase B) at a flow rate of 0.8 mL/min. The following program was used for the mobile phase: 0.8% to 2.4% phase B, 0–4.5 min; 2.4% to 4% phase B, 4.5–6 min; 4% to 6.8% phase B, 6–7.5 min; 6.8% to 14.4% phase B, 7.5–13 min; 14.4% to 15.4% phase B, 13–14 min; 15.4% to 24% phase B, 14–19 min; 24% to 40% phase B, 19–24 min; 40% phase B, 24–34 min; and 40% to 0.8% phase B, 34–40 min. The separation was carried out under room temperature and 150 bar pressure conditions. The detection was carried out in the wavelength range between 210 and 600 nm. Three wavelengths (240 nm, 280 nm, and 330 nm) were used for data analysis. Each extracted compound was identified by analysis of UV and MS data and quantified by DAD. For this quantification, a calibration curve of epigallocatechin (0.03–0.5 g/L), (−)-catechin hydrate (0.1–1.0 g/L), proanthocyanidin B-2 (0.03–1.0 g/L) taxifolin (0.06–1.0 g/L), 3,4 dihydroxybenzoic acid (0.12–1.2 g/L), (+)-epicatechin (0.1–1.06 g/L), quercetin (0.1–1.0 g/L), syringic acid (0.10–1.0 g/L), 3,4-dihydroxyphenyl acetic acid (0.10–1.0 g/L), *p*-hydroxybenzoic acid (0.11–1.06 g/L), and 2-(4-hydroxy-3-methoxy-phenyl) acetic acid (0.10–1.02 g/L) dissolved in methanol and filtered with a 0.2 µm filter was used.

#### 2.5.4. Release Study 

A release study was performed with the PE-loaded PCL/GEL electrospun nanofibers. In this test, each electrospun nanofiber mat was weighted accurately (3.0 mg) and incubated with 10 mL of PBS (1×, pH = 7.4) at 37 °C for 1, 3, 6, 24, 48, 72, 120, 168, 336, 540, and 840 h. After each time point, 500 µL of the sample was taken out to measure its absorbance using a UV-Vis spectrophotometer (Spectroquant Prove 600 spectrometer, Merck, Germany) at 281 nm based on a calibration curve (*R*^2^ = 0.9984). Then, 500 µL of fresh PBS was added to each nanofiber to ensure a constant volume during the assay. In addition, a blank absorbance (*A_o_*) and the maximum absorbance (*Abs_max_*) equivalent to the pure PE concentration loaded into the electrospun nanofibers were also measured. Finally, the content of PE released was calculated according to Equation (2):

(2) $$PE\;released\;percentage\;\left(\%\right)=\frac{Abs_{measured\;}-A_{o}}{Abs_{max}}*100$$

This test was performed in quintuplicate with reproducible results.

### 2.6. Antibacterial Assays

The antibacterial activity of PCL/GEL, PCL/GEL/0.18%PE, and PCL/GEL/0.36%PE electrospun nanofibers against *S. aureus* (Gram-positive) and *E. coli* (Gram-negative) was evaluated by a direct method. Each bacterial strain was separately incubated in a lysogeny broth medium (LB-medium) at 37 °C for 24 h. After that time, the optical density (OD) of each bacteria population was determined (600 nm, Thermo Scientific GENESYS 30; Schwerte; Germany) until reaching 0.015, according to the turbidity measurement of bacteria culture. Then, 10 mg of each electrospun nanofiber (sterilized by UV radiation for 1 h before the experiment) was placed in a 15 mL falcon tube with 30 µL of bacteria suspension and 2 mL of LB-medium. Each electrospun nanofiber was incubated at 37 °C for 3, 6, and 24 h. After each incubation time, the OD value of the samples was measured at 600 nm. The LB-medium and the PCL/GEL electrospun nanofiber mat were used as blank and control in this assay, respectively. Each measurement was performed in triplicate. The relative bacterial viability was calculated according to Equation (3):

(3) $$Relative\;bacterial\;viability\;\left(\%\right)=\frac{OD_{sample}}{OD_{control}}*100$$

### 2.7. Cell Culture

HaCaT cells were cultured in a DMEM supplemented with 10% fetal bovine serum, 4.5 g L^−1^ of glucose, and 1% penicillin/streptomycin solution at 37 °C for 24 h in a humidified atmosphere of 95% air and 5% CO_2_. After cells were grown to confluency, the DMEM was removed entirely and the cells were washed with PBS (5 mL). Then, the PBS was removed, and the cells were detached by trypsinization and counted by trypan blue assay using a hemocytometer (Roth, Germany). In parallel, each electrospun nanofiber mat was fixed on CellCrown 24 inserts (ScaffdexOy, Tampere, Finland) and sterilized by UV radiation for 1 h. Finally, counted cells were seeded on the top of electrospun nanofiber mats at a density of 125,000 cells/well and incubated at 37 °C in a humidified incubator with 5% CO_2_.

#### 2.7.1. Cell Viability Assay 

The cell viability of the electrospun nanofibers after 1- and 7-day incubation was analyzed using a WST-8 cell counting assay kit as indicated by the manufacturer. The absorbance of the dyes was measured in a spectrophotometric plate reader (FLUOstar-Omega BMG Labtech, Ortenberg, Germany) at 450 nm. In this assay, the PCL/GEL sample and the WST-8 reagent were used as control and blank, respectively. The cell viability of each electrospun nanofiber mat was calculated according to Equation (4):

(4) $$Cell\;viability\;\left(\%\right)=\frac{\left(Absorbance\;of\;sample-Absorbance\;of\;blank\right)}{\left(Absorbance\;of\;control-Absorbance\;of\;blank\right)}*100$$

#### 2.7.2. Cell Staining

HaCaT cells were stained with calcein, DAPI, and rhodamine phalloidin dyes after cell viability assays for cell distribution and cytoskeleton analysis by fluorescence microscopy (Axio Scope A1, Carl-Zeiss, Jena, Germany). Firstly, HaCaT cells were washed with PBS, fixed with 1 mL of calcein (4 µg mL^−1^), and incubated at 37 °C for 45 min in a 5% CO_2_ atmosphere. Afterward, HaCaT cells were washed with PBS to remove the calcein, and 1 mL of Fluoro-Fix was added for 15 min. Then, the cells were washed with PBS, and 1 mL of a permeabilization buffer solution was added for 5 min. After removing this solution, 1 mL of rhodamine phalloidin reagent (8 µg mL^−1^) was added to the cells, which were incubated at 37 °C for 45 min in 5% CO_2_. HaCaT cells were again washed with PBS and 1 mL of DAPI (1 µg mL^−1^) was added for 5 min, which was removed. Finally, HaCaT cells were stored at 4 °C with 1 mL of PBS until further analysis.

#### 2.7.3. SEM Analysis

After seven days of cell seeding, the morphology of HaCaT cells was analyzed by SEM, as mentioned in Section 2.5. In this process, after the cell medium was removed from each well, the electrospun nanofiber mats were rinsed with PBS. Afterward, the cells were first fixed with 2.5% (*v/v*) of glutaraldehyde solution for 2 h at room temperature, and then electrospun nanofibers were rinsed three times with PBS. Finally, the cell in the electrospun nanofibers was dehydrated in graded ethanol solutions (30, 50, 60, 70, 80, 90, 95, and 100%) and was dried with a critical point dryer (Leica EM CPD300, Istanbul, Turkey).

### 2.8. In Vitro Wound Healing Assay (Scratch Test) 

An in vitro wound healing assay was performed according to previously described experimental procedures [5,11], with slight modifications. Briefly, HaCaT cells (500,000 cells/well) were seeded into a 24-well plate and incubated at 37 °C for 24 h in a humidified atmosphere with 5% CO_2_. After that time, a vertical scratch was manually created in the middle of the HaCaT monolayer by using a 1000 µL sterile pipet tip. Then, each electrospun nanofiber mat was fixed on CellCrown 24 inserts (ScaffdexOy, Tampere, Finland) and placed on the 24-well plate without touching the surface. The wound closure rate and the cell migration were monitored over time (0, 4, 6, 24, 48, and 72 h) using a light microscope (Primo Vert, Carl Zeiss, Jena, Germany). Finally, the images were analyzed using ImageJ software. The wound closure rates were calculated according to Equation (5):

(5) $$Rate\;of\;wound\;closure\left(\%\right)=\frac{\left(A_{0}-A_{t}\right)}{A_{0}}*100$$

where *A*_0_ is the initial wound area and *A_t_* is the wound area after each time interval. All measurements were performed in triplicate. 

### 2.9. Statistical Analysis

Data analysis was performed using OriginPro8.5 software (Northampton, MA, USA), and ImageJ software was used to measure the average fiber diameters. Statgraphics Centurion XVII software (NIH, Bethesda, MD, USA) was used for one-way analysis of variance (ANOVA) and the analysis of multiple ranges (Duncan’s test). The level of significance was determined as *p* < 0.05, *p* < 0.01, and *p <* 0.001 in antibacterial activity and cell biocompatibility studies. The rest of the results were analyzed with a significance level of 95%. The data are presented as the mean ± SD, and the error bars are shown in each figure.

## 3. Results

### 3.1. Characterization of Electrospun Nanofibers

**Figure 1:**
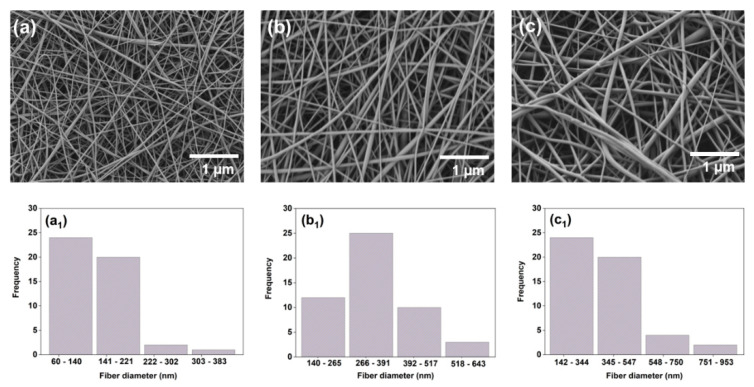
SEM images showing the morphology and the fiber diameter distribution of (**a**) PCL/GEL, (**b**) PCL/GEL/0.18%PE, and (**c**) PCL/GEL/0.36%PE electrospun nanofibers. The pore size distribution in each case was determined by ImageJ software and is shown at the bottom of the plots: (**a_1_**): PCL/GEL, (**b_1_**): PCL/GEL/0.18%PE, and (**c_1_**): PCL/GEL/0.36%PE electrospun nanofibers.

The surface morphology of PCL/GEL, PCL/GEL/0.18%PE, and PCL/GEL/0.36%PE electrospun nanofibers was investigated by SEM images. Figure 1 shows the formation of uniform, smooth, and bead-free electrospun nanofibers. Table 1 lists the average fiber diameters for each electrospun nanofiber. No statistical differences were observed in this parameter with PE incorporated into PCL/GEL electrospun nanofibers.

**Figure 2:**
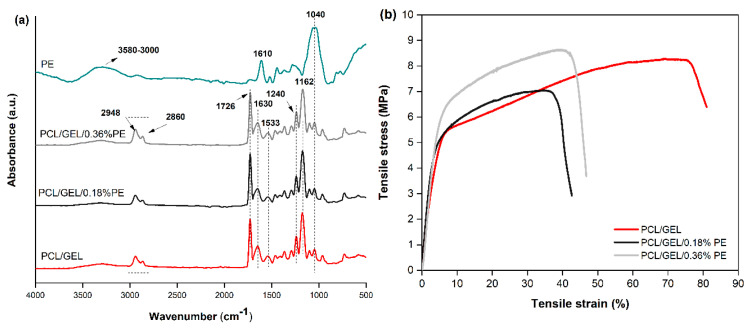
(**a**) ATR-FTIR spectra of PCL/GEL, PCL/GEL/0.18%PE, and PCL/GEL/0.36%PE electrospun nanofibers, also showing the spectrum of PE. (**b**) Tensile stress-strain curves of PCL/GEL, PCL/GEL/0.18%PE, and PCL/GEL/0.36%PE electrospun nanofibers.

The functional groups and the chemical bonds of the electrospun nanofibers and PE were investigated by ATR-FTIR spectroscopy (Figure 2a). All nanofiber’s spectra showed peaks at 2948 cm^−1^ and 2860 cm^−1^, corresponding to the asymmetric and symmetric stretching of CH_2_ bonds, which were associated with PCL. Moreover, a peak at 1726 cm^−1^, ascribed to carbonyl stretching, and the asymmetric and symmetric stretching of C-O-C bonds at 1240 cm^−1^ and 1162 cm^−1^ were observed in the electrospun nanofiber spectra [5,11,19]. Additionally, peaks at 1630 cm^−1^ and 1533 cm^−1^ were detected, which were associated with the amine I and N-H deformation for amide II of the GEL, respectively [5,28]. For the PE spectrum, peaks at 1040 cm^−1^ and 1610 cm^−1^ were observed, ascribed to the distinct functional groups of the polyflavonoids [29]. In addition, an OH^−^ band between 3580–3000 cm^−1^ was detected in PE-loaded electrospun nanofiber’s spectra. With the addition of PE, it was not possible to identify new peaks in the spectra of PE-loaded electrospun nanofibers compared to PCL/GEL. This could be due to the low PE concentration loaded in the PCL/GEL blend, which caused a prevalence of PCL and GEL bands in the ATR-FTIR spectra. 

TPC of PE-loaded PCL/GEL electrospun nanofibers was determined by the Folin–Ciocalteu assay, as described previously [27]. The results listed in Table 1 indicate TPC values of 8 ± 1 and 9 ± 1 mg equivalent gallic acid per gram of fiber for PCL/GEL/0.18%PE and PCL/GEL/0.36%PE electrospun nanofibers, respectively. No significant differences between both samples were observed due to the low PE concentration loaded into the PCL/GEL electrospun nanofibers. 

The wettability of the electrospun nanofibers was evaluated by contact angle measurements. The results listed in Table 1 exhibit hydrophilic surfaces for all electrospun nanofibers with values less than 90°. With the addition of 0.18% (*w/w*) of PE, the contact angle of nanofiber decreased by 55% compared to the PCL/GEL electrospun nanofiber mat, likely due to the interaction of PCL and GEL with the hydrophilic chain portions of the polyphenols. However, it was not possible to measure the contact angle of the PCL/GEL/0.36%PE electrospun nanofiber samples, possibly due to their high wettability. 

The influence of PE addition on the mechanical properties of PCL/GEL electrospun nanofiber mats was investigated by a uniaxial tensile test. Figure 2b shows the tensile stress-strain curves for each electrospun nanofiber mat, and the data of Young’s modulus, tensile strength, and elongation at break are listed in Table 2. No significant differences between Young’s modulus and elongation at break when PE was incorporated were observed. Conversely, the tensile strength values decreased for PE-loaded electrospun nanofiber compared to the PE-free PCL/GEL nanofiber. 

### 3.2. In Vitro Degradation Test 

**Figure 3:**
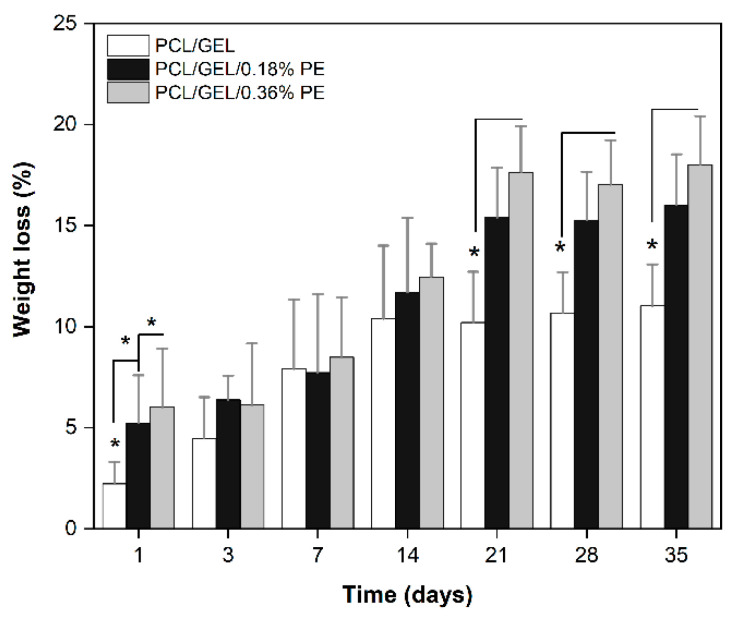
Weight loss of PCL/GEL, PCL/GEL/0.18%PE, and PCL/GEL/0.36%PE electrospun nanofibers over the 35 days of incubation in PBS (pH = 7.4, 37 °C). Data are shown as mean ± SD on triplicate experiments (*n* = 3, * *p* < 0.05).

The degradation behavior of the electrospun nanofibers was evaluated by monitoring their weight losses after 1, 3, 7, 14, 21, 28, and 35 days of incubation, as depicted in Figure 3. The results reveal a loss of weight over time for all electrospun nanofibers. As expected, the PCL/GEL electrospun nanofiber had the lowest weight loss over time, whereas an increment in the PE concentration increased the degradation rate of the electrospun nanofibers from the first day. No significant differences between the samples were observed between the 3rd and 14th days. In contrast, after days of incubation in PBS, in vitro degradation of PE-loaded electrospun nanofibers increased.

Additionally, the functional groups of the electrospun nanofibers were analyzed by ATR-FTIR spectroscopy at different time points during the in vitro degradation assay (Figure S1). The FTIR spectra showed a decrease in the intensity of the characteristic peaks of PCL, GEL, and PE compared to the original FTIR spectra (Figure 2a). In addition, the -OH band belonging to the PE spectrum became broader and more noticeable in the spectra obtained between 14 and 21 days of incubation for electrospun nanofibers containing 0.18% PE. Similarly, the -OH band intensity increased for the electrospun nanofiber loaded with 0.36% PE between the 7th and 28th days. The appearance of new peaks in these spectra was not observed. 

### 3.3. Phenol Composition of PE

The phenol composition of PE was determined with a reversed-phase high-performance chromatography (RP-HPLC)-mass spectrometry (MS) system coupled with a diode array detector (DAD). Table S1 lists the names and the content of each of the compounds identified in the chemical composition of PE. Twelve compounds were detected, with a predominance of (−)-catechin and taxifolin of more than 50% of their content compared to the other compounds. These results are consistent with previous studies demonstrating the presence of most of the identified compounds in the composition of *Pinus radiata* [27], except for tentative compounds. However, the absence of epigallocatechin in our extracts is contradictory to previously reported results [29]. 

### 3.4. In Vitro Release of PE

**Figure 4:**
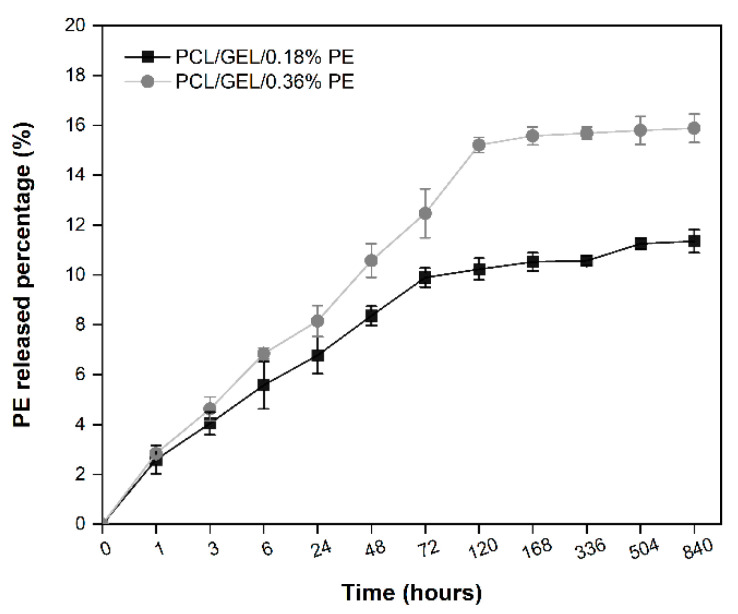
Release profiles over time of PE from PCL/GEL electrospun nanofibers immersed in PBS (pH = 7.4, 37 °C).

The content PE released from PCL/GEL/0.18%PE and PCL/GEL/0.36%PE electrospun nanofibers was evaluated over time using a PBS medium at physiological conditions to simulate the wound environment. Figure 4 shows the release profiles of electrospun nanofibers for both PE concentrations. The results indicate that the PE-loaded PCL/GEL electrospun nanofibers have a triphasic release profile. Firstly, nanofibers showed gradually increased PE release up to 1 h, then a burst release up to 72 h, followed by the plateau stage from 120 to 840 h. At 120 h, the PE release rate was 10% and 15% for electrospun nanofibers loaded with 0.18% and 0.36% of PE, respectively. Both profiles maintained an almost steady concentration of about 11% and 16% for PCL/GEL/0.18%PE and PCL/GEL/0.36%PE electrospun nanofibers in the final phase, respectively. 

### 3.5. Antibacterial Activity of Electrospun Nanofibers

**Figure 5:**
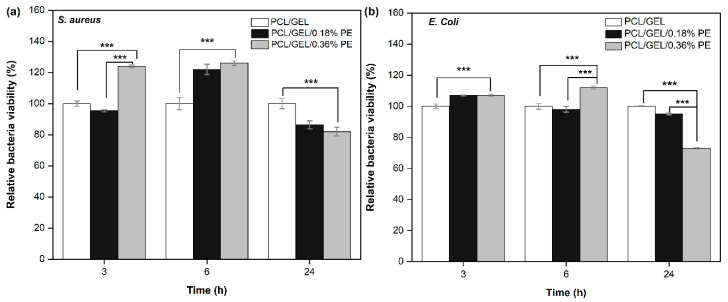
Antibacterial activity of PCL/GEL, PCL/GEL/0.18%PE, and PCL/GEL/0.36%PE electrospun nanofibers after 3, 6, and 24 h of incubation period: (**a**) *S. aureus* bacteria and (**b**) *E. coli* bacteria (Duncan test by one-way ANOVA analysis, *n* = 3, *** *p* < 0.001).

The antibacterial activity of PCL/GEL, PCL/GEL/0.18%PE, and PCL/GEL/0.36%PE electrospun nanofibers against *S. aureus* (Gram-positive) and *E. coli* (Gram-negative) bacteria was evaluated (Figure 5). The results reveal that the addition of two different concentrations of PE into PCL/GEL electrospun nanofibers increased the *S. aureus* bacteria activity during the first 6 h of incubation. In contrast, increasing PE concentration in the electrospun nanofibers decreased the *E. coli* bacteria viability after 6h of incubation, reaching inhibition percentages of 6 ± 0.1% and 23 ± 0.3% for PCL/GEL/0.18%PE and PCL/GEL/0.36%PE electrospun nanofibers, respectively. Similarly, inhibition percentages of 14 ± 0.1% and 18 ± 0.2% against *S. aureus* were reached with the electrospun nanofibers loaded with 0.18% and 0.36% PE, respectively. Consequently, the PCL/GEL/0.36%PE electrospun nanofiber was found to result in the lowest bacterial viability, around 73% against *E. coli* bacteria. In addition, the PE-loaded PCL/GEL electrospun nanofibers start to reduce bacterial viability after 24 h incubation for both bacteria strains, which could be attributed to their release profiles, as discussed in Section 3.3. The lower effect on the bacterial viability of PE-loaded electrospun nanofibers could be due to the limited PE concentration in the nanofiber. This phenomenon can also be explained by the high release profile of the PCL/GEL/0.36%PE electrospun nanofiber.

### 3.6. In Vitro Cell Viability Assay

**Figure 6:**
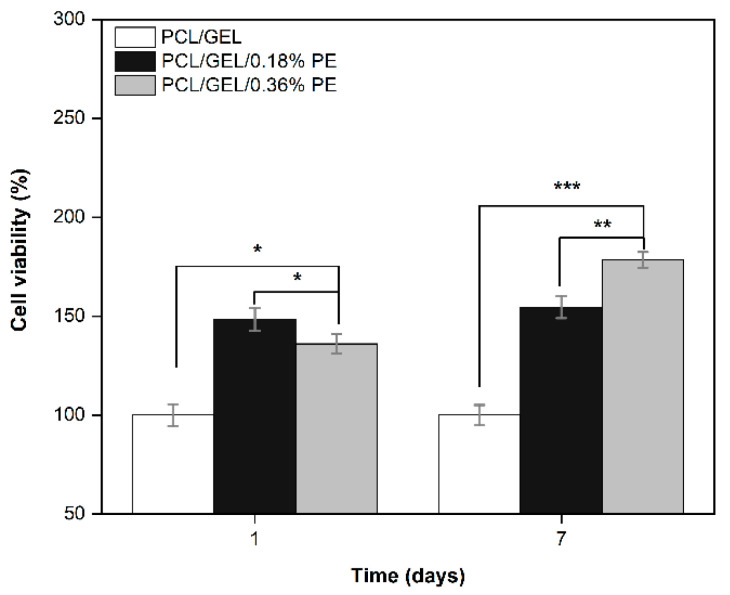
HaCaT cell viability onto the surfaces of PCL/GEL, PCL/GEL/0.18%PE, and PCL/GEL/0.36%PE electrospun nanofiber mats after 1 and 7 days of incubation (*n* = 3, * *p* < 0.05, ** *p* < 0.01, *** *p* < 0.001).

The biocompatibility of the electrospun nanofibers was evaluated by an in vitro cell viability study on HaCaT cells (Figure 6). An increase in the cell viability associated with PE-loaded electrospun nanofibers compared to PCL/GEL nanofiber after 1 day was observed. However, after 7 days of incubation, only the PCL/GEL/0.36%PE electrospun nanofiber increased the proliferation of HaCaT cells. Moreover, the results indicated that increasing the PE content increases the viability of the HaCaT cells. This could be attributed to a higher PE release from the PCL/GEL/0.36%PE nanofiber and also to the chemical composition of PE containing proanthocyanidins (PAs) and a high polyphenol content, as explained below. 

**Figure 7:**
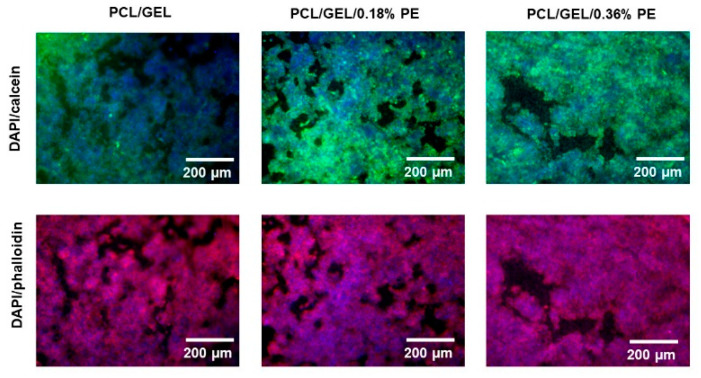
Fluorescent images of HaCaT cells cultured on the surfaces of PCL/GEL and PE-loaded PCL/GEL electrospun nanofiber mats after staining with DAPI–calcein and DAPI–phalloidin. The cytoplasm of cells, nuclei, and cytoskeleton are stained in green, blue, and red, respectively.

On the other hand, HaCaT cells cultured on electrospun nanofiber surfaces were examined under a fluorescence microscope. Figure 7 shows the fluorescence images of HaCaT cells seeded on the electrospun nanofiber mats after being stained with DAPI-calcein and DAPI–phalloidin. For this purpose, the DAPI-calcein staining was used to analyze the cytoplasm of live cells (green) and cell nuclei (blue), whereas DAPI-phalloidin was used to investigate the cell nuclei (blue) and the presence of cell cytoskeleton protein (red). The images show that HaCaT cells were well spread throughout the matrix in all electrospun nanofiber mats. Additionally, it was confirmed that the incorporation of PE into PCL/GEL electrospun nanofibers had no toxic effect on HaCaT cells.

Likewise, the morphology of HaCaT cells after 7 days of incubation with electrospun nanofibers was observed by SEM to analyze cell attachment (Figure S2). The images show the formation of several portions of cell colonies attached to the electrospun nanofiber mats, which were well entangled and spread within them. These findings are in agreement with the fluorescence images; therefore, cell growth and proliferation of HaCaT cells on the electrospun nanofiber’s surfaces were confirmed. 

### 3.7. In Vitro Wound Healing Assay (Scratch Test)

**Figure 8:**
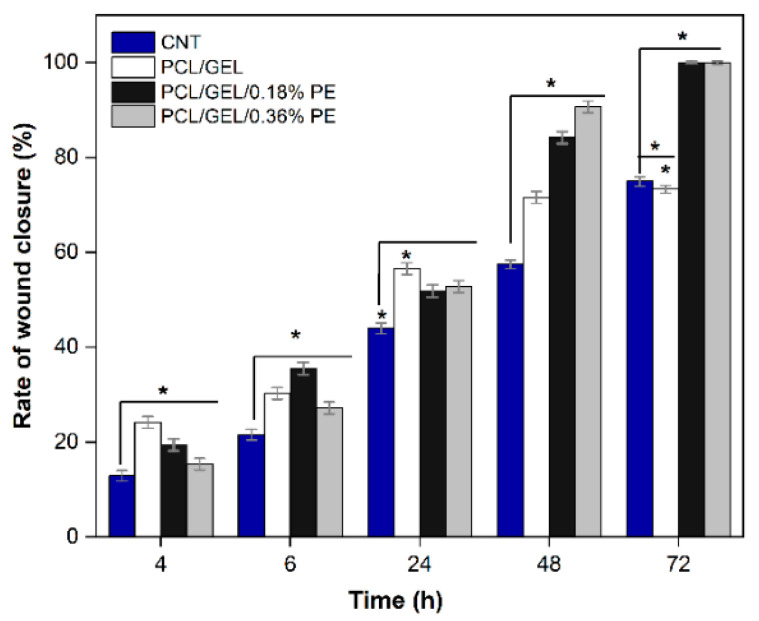
In vitro wound healing assay over time for PCL/GEL, PCL/GEL/0.18%PE, and PCL/GEL/0.36%PE electrospun nanofiber mats. The CNT used in this assay corresponded to HaCaT cells without a sample. The asterisk (*) indicates a significant difference (* *p* < 0.05) when analyzed by Duncan test by one-way ANOVA analysis.

To better evaluate the potential of the electrospun nanofibers for wound healing applications, an in vitro wound healing assay (scratch) using HaCaT cells was performed. In this test, it was assumed that HaCaT cells attempted to migrate along the edges of the scratch zone to establish cell-cell contact, which led to the closure of the wound. Both cell migration and wound closure rate were monitored over time (Figure S3 and Figure 8, respectively). The optical microscopy images show that HaCaT cells migrated to the scratch zone after 72 h incubation when in contact with PE-loaded PCL/GEL electrospun nanofibers. These results were confirmed by the determined wound closure rates (Figure 8). Similarly, the PE-loaded electrospun nanofibers increased the wound closure rate compared to the PCL/GEL nanofiber. After 72 h of treatment, the PE-loaded PCL/GEL electrospun nanofiber mats achieved a complete wound closure, whereas the PCL/GEL electrospun nanofiber mats and the control (CNT) reached 75 ± 1% and 73 ± 1% of wound closure, respectively. These findings are in agreement with the cell viability results, showing that PE-loaded PCL/GEL electrospun nanofiber mats enhanced the viability and migration of the HaCaT cells.

## 4. Discussion

The combination of natural compounds with engineered biomaterials to produce new composites is of increasing interest in the biomedical field. Among these, *Pinus radiata* bark extracts, rich in phenolic compounds, particularly in flavonoids, possess interesting antioxidant and anti-inflammatory activities that could support their therapeutic use in biomedical applications. The combination of *Pinus radiata* bark extracts with polymeric matrices has also proven to be a promising strategy to counteract potential drawbacks (mentioned earlier) of polyphenols [5,10,15,30], thus helping to preserve and protect their functionalities [31]. In this context, the mixture of these materials could also provide ideal protection for PE against external factors such as light, heat, and humidity, thus preventing easy oxidation of its components and improving its controlled release. This study investigates the potential of PCL/GEL and PE-loaded PCL/GEL electrospun nanofibers fabricated via electrospinning for wound healing applications.

Electrospinning is a well-established technique widely used for the fabrication of nanofibrous mats for numerous biomedical applications [19]. Several parameters such as concentration and viscosity of the polymer solution, electrical conductivity, temperature, humidity, voltage, and tip-to-collector distance influence the electrospinning process [10,32,33]. In addition, decreasing the electrical conductivity or increasing the solution viscosity can increase the average fiber diameter [5,34,35], thus modifying the electrospun sample’s morphology. The combined action of the above factors generates a direct effect on the surface morphology of the electrospun samples. Using SEM imaging, we observed that the addition of PE into PCL/GEL electrospun nanofibers did not affect the surface morphology or the average fiber diameter due to the limited PE concentration in the electrospun nanofibers. This finding is favorable for wound healing applications because it allows the development of a robust method for the reproducible fabrication of electrospun samples. 

The influence of the electrospinning parameters on the morphological characteristics of PCL/GEL nanofibers loaded with different bioactive compounds has been previously investigated. Jiang et al. [36] reported an increase in the average fiber diameter for PCL/GEL nanofibers loaded with palmatine compared to PCL/GEL nanofibers due to the decrease in the electrical conductivity of the spinning solution by adding palmatine. Similarly, Adeli-Sardou et al. [37] found that the average fiber diameter of PCL/GEL nanofibers loaded with lawsone increased compared to that of PCL/GEL nanofibers. Unalan et al. [5] reported as well that the increase in the fiber diameter of CLV-loaded PCL/GEL fiber mats compared to PCL/GEL fiber mats could be attributable to a reduction in solution electrical conductivity. These studies demonstrated that the morphological properties of electrospun samples are modified by loading natural compounds into the starting PCL/GEL solution. Therefore, future morphological studies should be performed in the PE-loaded PCL/GEL electrospun nanofibers using higher PE concentrations to examine possible morphological changes after adding PE.

The chemical bonds of electrospun nanofibers and PE were confirmed through ATR-FTIR spectroscopy. The TPC was measured for the two PE-loaded electrospun nanofibers through a Folin-Ciocalteu assay. Both analyses confirmed the successful loading of PE into the PCL/GEL electrospun nanofibers.

The wettability of electrospun nanofiber mats is an essential property for biomedical applications since they should absorb wound exudates and maintain moisturized environments for wound healing [5,15,38]. When the hydrophilicity was measured, it was found that the contact angle for the PCL/GEL/0.18%PE electrospun nanofiber significantly decreased compared to the PCL/GEL sample. This result could be attributed to the presence of -OH groups and hydrophilic chain portions contained in the PE’s chemical structure, as shown in the ATR-FTIR analysis. According to Ramalingam et al. [20], the wettability of the PCL/GEL mats increased after adding *Gymnema sylvestre* leaf extracts compared to the PCL/GEL nanofiber’s wettability due to the presence of multiple -OH groups in the extract’s structure. In another study, Unalan et al. [5] ascribed the decrease in the contact angle for CLV-loaded PCL/GEL fiber mats compared to the PCL/GEL fiber mat’s wettability to the presence of polar and hydrophilic functional groups in the CLV structure. Both studies coincide in the fact that the presence of hydrophilic groups from new bioactive compounds increases the wettability of the electrospun samples, as demonstrated in the present study. Thus, PE-loaded electrospun nanofibers could be useful for wound healing applications due to their hydrophilic nature.

The mechanical properties of nanofibrous materials also play an essential role in wound healing applications since the nanofibers must be strong enough to withstand the mechanical stresses applied without causing large deformation or fracture during wound healing [20,37]. In the current study, the tensile strength values of fiber mats declined when the PE content loaded into the PCL/GEL electrospun nanofibers increased. Our results also agree with previous studies demonstrating that loading plant extracts in electrospun nanofibers reduces the mechanical performance of nanofibrous materials [37,38,39]. Several explications have been attributed to the reduction in the mechanical properties of fibrous materials after loading natural compounds. Salehi et al. [39] associated this behavior with the formation of random fibers instead of aligned fibers. On the other hand, Sardou et al. [37] ascribed this reduction to the plasticizing effect of these materials. In contrast, Mohamadi et al. [21] stated that after loading natural compounds into nanofibers, semi-interpenetrated systems are formed, leading to a decrease in the mechanical properties of the polymeric systems. In all these cases, there is a reduction in the cross-sectional area per unit area of the fibers, resulting in the formation of nanofiber networks capable of resisting external tensile forces due to their random distribution. In contrast, Young’s modulus and elongation at break were not affected after adding PE into electrospun nanofibers. Overall, the measured mechanical property values are within the ranges reported by other authors for wound dressing applications [37,40].

The biodegradability of materials is a critical requirement that must be evaluated for wound healing applications. In the present study, we found that PE-loaded electrospun nanofibers exhibited higher degradation rates over time compared to the PCL/GEL nanofiber. This degradation behavior could be mainly due to the presence of hydrophilic chain portions and -OH groups in the PE structure, enhancing the affinity of PE-loaded electrospun nanofibers for water molecules. These results can be further explained by the PE release, which will be discussed below. These findings also agree with our wettability data. By monitoring the electrospun nanofiber’s functional groups during the degradation assay, slight changes in the -OH band intensity were detected. This result might be attributable to the intermolecular interactions occurring between the electrospun nanofibers and PBS, which are enhanced for PE-loaded electrospun nanofibers due to the presence of PE hydroxylic groups. In contrast, no noticeable changes in the main functional groups of GEL and PCL were detected during degradation. Due to the chemical complexity of these molecules, the knowledge about the nature of the degradation products is still limited. To our knowledge, there are no previous studies in which the degradation products of *Pinus radiata* bark extracts have been isolated and characterized the mechanism of degradation successfully elucidated. 

The degradation behavior of natural extracts loaded into PCL/GEL electrospun nanofibers has been investigated in previous studies, showing that the incorporation of natural compounds into PCL/GEL nanofibers increases the degradation rates over time [10,15,22,36,37], which agrees with our degradation results. In addition, two of these studies support the idea that the degradation rate increases due to lowering intermolecular forces between PCL/GEL nanofibers after the loading of extracts [10,37]. Based on our degradation results, PE-loaded electrospun nanofibers could be beneficial for wound healing applications because of their combination of biodegradable and hydrophilic properties. 

In vitro drug release testing is required to evaluate a material’s applicability in treating different wounds. In the present study, the process of releasing PE from PE-loaded electrospun nanofibers exhibited a sustained and controlled release kinetics, which is favorable for wound healing applications. The low release percentages achieved by both PE-loaded electrospun nanofibers over time could be explained, firstly, by the limited PE concentration loaded into the PCL/GEL electrospun nanofiber, which is consistent with the TPC results. Secondly, the low PE release may be due to its own nature, including its complex chemical structure that consists of several compounds, as summarized in Table S1. In addition, these results are directly related to the degradation behavior of our electrospun nanofibers since, due to the high susceptibility of phenolic compounds to degradation by many environmental factors [41,42], it is difficult to identify which small molecules are diffusing out and releasing into the medium. Consequently, different release and degradation rates are expected for each of the compounds present in PE. 

In addition, it is worth mentioning that the release processes of molecules loaded into nanofibers are influenced by the hydrophilic/hydrophobic nature of the drug, the morphology, the pore size of the nanofibers, and the diffusive processes involved during the release, among other factors [15,43]. The combined action of such variables directly influences the release of compounds loaded into electrospun fibrous samples. 

The release of phenolic compounds loaded into nanofibrous materials has been previously investigated by other authors. Lin et al. [43] reported that grape seed extract (GSE)-loaded silk fibroin(SF)/polyethylene oxide (PEO) nanofibers released 65% of the GSE content after 350 h in PBS. In a similar study, Locilento et al. [15] found that polylactic acid (PLA)/PEO and PLA nanofibrous membranes loaded separately with GSE released about 40% and 70% of their GSE content after 700 h of testing in a PBS solution, respectively. Both studies agreed on loading GSE with a chemical composition similar to PE into nanofibrous matrices. Therefore, the differences in the released extract contents from nanofibers could be attributed to the extract’s content loaded into the nanofiber. Due to the structural complexity of PE, it is difficult to measure its release accurately; therefore, further studies in this area are required.

Antibacterial activity of wound dressing materials plays a crucial role in avoiding bacterial contamination in wound healing processes [37]. Among the microorganisms involved in such processes are *S. aureus* bacteria, which appear at an early stage of healing, and *E. coli* bacteria, which are more related to chronic wounds [11]. In previous studies, catechin and taxifolin, as the main compounds of PE, were investigated in terms of their antibacterial activity. For instance, Díaz-Gómez et al. [44] reported that the inhibition zone was 5 mm for 7.5 mg catechin, and the inhibition zone increased with increasing catechin concentration, indicating the inhibitory effect against *E. coli*. In another study, Ahamad et al. [45] investigated the antibacterial activity of taxifolin toward *S. mutants* and *L. acidophilus.* Their results revealed that the inhibition zone was 18–22 mm for *S. mutans* and 7–13 mm for *L. acidophilus* at concentrations in the range of 1.5–2.5 mg/mL. The mentioned studies thus confirmed that PE has the potential to be used as antibacterial material. In the present study, the antibacterial activity of PE-loaded PCL/GEL electrospun nanofibers was tested on *S. aureus* and *E. coli* bacteria. A slight bacterial viability reduction in both bacteria strains was observed after 24 h incubation, being higher for the PCL/GEL/0.36%PE electrospun nanofiber against *E. coli* bacteria. This finding is related to a higher content of PE released by the PCL/GEL/0.36%PE nanofiber at 24 h and could be also ascribed to the different characteristics and morphology of each bacteria strain. Gram-negative bacteria have a higher resistance to being penetrated due to their double-layer cell membrane compared to the single membrane of Gram-positive bacteria [5,46]. In addition, the antibacterial properties of phenolic compounds such as GSE, with a chemical structure similar to PE, have been previously investigated using various bacteria strains. GSEs have shown suitable antibacterial properties against *A. actinomycetemcomitans*, *S. mutans,* and *E. faecalis* [47,48]. In addition, they have exhibited inhibitory effects on *E. coli* and *S. aureus* [49]. The GSE antibacterial effects have been attributed by some authors to the general mechanism of polyphenols acting on the bacterial cell membrane [48]. However, other authors have associated it with the presence of gallic acid that had shown inhibitory effects on *E. coli* and *S. enteritidis* [50]. Based on these results, future investigations by using higher PE concentrations should be performed to verify the antibacterial activity of PE-loaded PCL/GEL electrospun nanofibers.

Keratinocyte cell lines from adult human skin (HaCaT cells) have been extensively used in scientific research as a reproducible model to characterize skin keratinocytes in vitro due to their high capacity to differentiate and proliferate in vitro [51,52,53]. Therefore, HaCaT cells were used in this research as an in vitro model representative for human skin to evaluate the biocompatibility of the PE-loaded electrospun nanofibers. In this regard, an in vitro cell viability assay was performed by directly seeding the cells on the electrospun nanofibers to investigate cell adhesion, migration, and proliferation behavior for an incubation period of up to 7 days. HaCaT cells exhibited increased viability on electrospun nanofibers containing PE from the first day, which is correlated with the release profile of PE (see Figure 4). Additionally, the viability of PCL/GEL/0.36%PE nanofibers significantly increased after seven days of incubation compared to day 1, indicating HaCaT cell proliferation. On the other hand, our experiments have shown that both samples were non-toxic for wound healing applications, and cell proliferation was induced by PCL/GEL/0.36%PE electrospun nanofibers after 7-day incubation. These results agree with the PE release profiles showing that PCL/GEL/0.36%PE nanofibers released more PE than PCL/GEL/0.18%PE nanofibers after incubation of up to 7 days, which could explain the higher cell viability in the PCL/GEL/0.36%PE electrospun nanofiber. In addition to cell viability investigation, the material’s biocompatibility is related to the cell adhesion ability on the material surface. HaCaT cells were well spread throughout the matrix of all electrospun nanofiber mats and attached to their surfaces, which is consistent with the wettability results because materials with high hydrophilicity provide better cell growth, attachment, and proliferation environments. All these findings can be explained, firstly, by the presence of GEL in the electrospun nanofibers, which contain the major components of the extracellular matrix that promote cell attachment and proliferation [54]. Secondly, the PE incorporated into electrospun nanofibers improved the in vitro biocompatibility of HaCaT cells, which might be ascribed both to the high polyphenol concentration and to the presence of PAs in the chemical composition of PE, as reported in previous studies [15,55,56]. In a related study, Locilento et al. [15] demonstrated an enhancement in the activity of human foreskin fibroblast (HFF1) cells for GSE-loaded PLA/PEO nanofibrous membranes compared to a pristine PLA nanofiber. Their result was ascribed to the presence of PAs in the chemical composition of GSE, which is also present in PE’s chemical composition. In addition, the authors demonstrated that HFF1 cells were able to attach and grow on the nanofibrous membranes containing GSE [15]. Herein, in vitro cell investigation demonstrated that PE-loaded PCL/GEL electrospun nanofibers improved cell biocompatibility, which suggests their promising potential for wound healing applications. 

Wound healing is a complex process involving an initial inflammatory phase, a proliferative/repair phase, and a remodeling phase [1]. In our study, the in vitro wound healing assay results showed that PE has a potential effect on wound healing considering that PE-loaded electrospun nanofibers favored the HaCaT cells’ migration to the scratch area after 72 h treatment, thus allowing the complete wound closure. These findings agree with the HaCaT cell viability results, demonstrating that the PE-loaded PCL/GEL electrospun nanofibers lead to improved cell biocompatibility. In the literature, studies conducted on evaluating the cell migration effect in natural compounds are limited. Among them, Schuhladen et al. [11] demonstrated that adding Manuka honey and borate bioactive glass into PCL nanofibers improves HaCaT cell migration compared to neat PCL fibers. Similarly, Unalan et al. [5] demonstrated that normal human dermal fibroblast (NHDF) cell migration and proliferation were reduced by increasing CLV concentration in PCL/GEL fiber mats, although no adverse effects on cell viability were observed. According to the authors’ knowledge, this is the first study to evaluate the HaCaT cell migration cultured in PE-loaded PCL/GEL electrospun nanofibers to determine the wound closure rate. Given the positive results obtained, further studies should be conducted to assess the biological activity of these electrospun nanofibers using higher PE concentrations to verify this effect, also for longer periods of time. 

The combination of natural compounds with engineered biomaterials has emerged as a promising approach in the biomedical field [57]. The complex chemical structures of such compounds, including their different main compounds and functional groups, directly influence their physicochemical and biological performance. In the present study, the formation of nanofibrous structures with outstanding cell biocompatibility and remarkable potential for usage in wound healing applications was highlighted.

## 5. Conclusions

In this study, biocompatible and biodegradable PCL/GEL electrospun nanofibers loaded with different concentrations of PE were successfully fabricated via electrospinning. Smooth, uniform, and bead-free surfaces were observed for all electrospun nanofibers. The PE addition did not affect the morphology or the average diameter of electrospun nanofibers. However, the wettability of the electrospun nanofibers was enhanced with the loading of PE, whereas the tensile strength values were reduced. PE-loaded electrospun nanofibers also exhibited a higher degradation rate over time compared to PE-free PCL/GEL nanofibers. Moreover, PE was successfully released from the fibrous mats in a rather controlled fashion. No significant bacteriostatic and bactericidal effects were observed for both PE-loaded PCL/GEL electrospun nanofibers when the antibacterial activity against *S. aureus* and *E. coli* bacteria was evaluated. Interestingly, PE-loaded electrospun nanofibers enhanced the growth, attachment, and proliferation of HaCaT cells, as well as favoring cell migration towards the scratch area during the in vitro wound healing assay. Consequently, this study’s findings provide evidence supporting that PE-loaded PCL/GEL electrospun nanofibers are promising candidates for wound healing usage, enlarging the family of phytotherapeutic agents containing electrospun fibers for such application. For this purpose, further investigations such as wound healing in vivo studies and analysis related to the antibacterial, antioxidant, and anti-inflammatory activity of PE-loaded PCL/GEL electrospun nanofibers should be performed. 

## Figures and Tables

**Table 1:** Values of average fiber diameter, TPC, and contact angle of electrospun nanofiber mats.

Sample Code	Average Fiber Diameter (nm)	TPC *	Contact Angle (°)
PCL/GEL	159 ± 53	-	36 ± 7
PCL/GEL/0.18%PE	343 ± 116	8 ± 1	20 ± 8
PCL/GEL/0.36%PE	388 ± 147	9 ± 1	n.d.

* TPC is expressed as mg of equivalent gallic acid/mg of fiber, n.d.: not detected.

**Table 2:** Mechanical properties of electrospun nanofiber mats.

Samples	Young’s Modulus (MPa)	Tensile Stress (MPa)	Elongation at Break (%)
PCL/GEL	191 ± 64	20 ± 4 ^a^	81 ± 23
PCL/GEL/0.18%PE	151 ± 23	14 ± 5 ^ab^	49 ± 9
PCL/GEL/0.36%PE	144 ± 54	8 ± 2 ^b^	57 ± 16

The letters a and b indicate significant statistical differences for *p*-value < 0.05.

## Data Availability

Not applicable.

## Supplementary Materials

The following supporting information can be downloaded at: https://www.mdpi.com/article/10.3390/polym14122331/s1, The Supporting Information contains information about the FTIR spectra of electrospun nanofibers after degradation, the phenol composition and content of the *Pinus radiata* bark extract, and SEM images of HaCaT cells cultured on nanofiber’s surfaces. The micrographs of HaCaT cell migration during the wound healing assay are also included in this document. Figure S1: FTIR spectra obtained during the in vitro degradation assay of (a) PCL/GEL, (b) PCL/GEL/0.18%PE, and (c) PCL/GEL/0.36%PE electrospun nanofiber mats; Figure S2: SEM micrographs of HaCaT cells cultured on (a) PCL/GEL, (b) PCL/GEL/0.18%PE, and (c) PCL/GEL/0.36%PE electrospun nanofiber mats. The images were taken after incubation and staining studies; Figure S3: Micrographs of the cell migration into a scratch area over a 72 h period in CNT, PCL/GEL, PCL/GEL/0.18%PE, and PCL/GEL/0.36%PE electrospun nanofiber mats; Table S1: Phenol composition and content of the *Pinus radiata* bark extract.

## References

[1] Zheng L., Zhang S., Ying Z., Liu J., Zhou Y., Chen F. (2020). Engineering of Aerogel-Based Biomaterials for Biomedical Applications. Int. J. Nanomed..

[2] Deng P., Jin W., Liu Z., Gao M., Zhou J. (2021). Novel multifunctional adenine-modified chitosan dressings for promoting wound healing. Carbohydr. Polym..

[3] Sezer U.A., Kocer Z., Aru B., Demirel G.Y., Gulmez M., Aktekin A., Ozkara S., Sezer S. (2016). Combination of gelatin and tranexamic acid offers improved haemostasis and safe use on internal hemorrhage control. RSC Adv..

[4] Feng C., Li J., Wu G.S., Mu Y.Z., Kong M., Jiang C.Q., Cheng X.J., Liu Y., Chen X.G. (2016). Chitosan-Coated Diatom Silica as Hemostatic Agent for Hemorrhage Control. ACS Appl. Mater. Interfaces.

[5] Unalan I., Endlein S.J., Slavik B., Buettner A., Goldmann W.H., Detsch R., Boccaccini A.R. (2019). Evaluation of Electrospun Poly(ε-caprolactone)/Gelatin Nanofiber Mats Containing Clove Essential Oil for Antibacterial Wound Dressing. Pharmaceutics.

[6] Agarwal T., Narayan R., Maji S., Behera S., Kulanthaivel S., Maiti T.K., Banerjee I., Pal K., Giri S. (2016). Gelatin/Carboxymethyl chitosan based scaffolds for dermal tissue engineering applications. Int. J. Biol. Macromol..

[7] Anjum S., Arora A., Alam M., Gupta B. (2016). Development of antimicrobial and scar preventive chitosan hydrogel wound dressings. Int. J. Pharm..

[8] Ma R., Wang Y., Qi H., Shi C., Wei G., Xiao L., Huang Z., Liu S., Yu H., Teng C. (2019). Nanocomposite sponges of sodium alginate/graphene oxide/polyvinyl alcohol as potential wound dressing: In vitro and in vivo evaluation. Compos. Part B Eng..

[9] Rezaii M., Oryan S., Javeri A. (2019). Curcumin nanoparticles incorporated collagen-chitosan scaffold promotes cutaneous wound healing through regulation of TGF-β1/Smad7 gene expression. Mater. Sci. Eng. C.

[10] Unalan I., Slavik B., Buettner A., Goldmann W.H., Frank G., Boccaccini A.R. (2019). Physical and Antibacterial Properties of Peppermint Essential Oil Loaded Poly(ε-caprolactone) (PCL) Electrospun Fiber Mats for Wound Healing. Front. Bioeng. Biotechnol..

[11] Schuhladen K., Raghu S.N.V., Liverani L., Neščáková Z., Boccaccini A.R. (2020). Production of a novel poly(ε-caprolactone)-methylcellulose electrospun wound dressing by incorporating bioactive glass and Manuka honey. J. Biomed. Mater. Res. Part B Appl. Biomater..

[12] Zhang Y., Song W., Lu Y., Xu Y., Wang C., Yu D.-G., Kim I. (2022). Recent Advances in Poly(α-*L*-glutamic acid)-Based Nanomaterials for Drug Delivery. Biomolecules.

[13] Howell-Jones R.S., Wilson M.J., Hill K.E., Howard A.J., Price P.E., Thomas D. (2005). A review of the microbiology, antibiotic usage and resistance in chronic skin wounds. J. Antimicrob. Chemother..

[14] Rivadeneira J., Di Virgilio A.L., Audisio M.C., Boccaccini A.R., Gorustovich A.A. (2016). 45S5 Bioglass^®^ concentrations modulate the release of vancomycin hydrochloride from gelatin–starch films: Evaluation of antibacterial and cytotoxic effects. J. Mater. Sci..

[15] Locilento D.A., Mercante L.A., Andre R.D.S., Mattoso L.H.C., Luna G.L.F., Brassolatti P., Anibal F.D.F., Correa D.S. (2019). Biocompatible and Biodegradable Electrospun Nanofibrous Membranes Loaded with Grape Seed Extract for Wound Dressing Application. J. Nanomater..

[16] Mellado C., Figueroa T., Báez R., Castillo R., Melendrez M., Schulz B., Fernández K. (2018). Development of Graphene Oxide Composite Aerogel with Proanthocyanidins with Hemostatic Properties As a Delivery System. ACS Appl. Mater. Interfaces.

[17] Kalkhoran A.H.Z., Naghib S.M., Vahidi O., Rahmanian M. (2018). Synthesis and characterization of graphene-grafted gelatin nanocomposite hydrogels as emerging drug delivery systems. Biomed. Phys. Eng. Express.

[18] Tracy L.E., Minasian R.A., Caterson E. (2016). Extracellular Matrix and Dermal Fibroblast Function in the Healing Wound. Adv. Wound Care.

[19] Liverani L., Boccaccini A.R. (2016). Versatile Production of Poly(Epsilon-Caprolactone) Fibers by Electrospinning Using Benign Solvents. Nanomaterials.

[20] Ramalingam R., Dhand C., Leung C.M., Ezhilarasu H., Prasannan P., Ong S.T., Subramanian S., Kamruddin M., Lakshminarayanan R., Ramakrishna S. (2019). Poly-ε-Caprolactone/Gelatin Hybrid Electrospun Composite Nanofibrous Mats Containing Ultrasound Assisted Herbal Extract: Antimicrobial and Cell Proliferation Study. Nanomaterials.

[21] Mohamadi P.S., Hivechi A., Bahrami H., Hemmatinegad N., Milan P.B. (2021). Antibacterial and biological properties of coconut oil loaded poly(ε-caprolactone)/gelatin electrospun membranes. J. Ind. Text..

[22] Chong L.H., Lim M.M., Sultana N. (2015). Fabrication and Evaluation of Polycaprolactone/Gelatin-Based Electrospun Nanofibers with Antibacterial Properties. J. Nanomater..

[23] Schuhladen K., Roether J.A., Boccaccini A.R. (2019). Bioactive glasses meet phytotherapeutics: The potential of natural herbal medicines to extend the functionality of bioactive glasses. Biomaterials.

[24] Bocalandro C., Sanhueza V., Gómez-Caravaca A.M., González-Álvarez J., Fernández K., Roeckel M., Rodríguez-Estrada M.T. (2012). Comparison of the composition of *Pinus radiata* bark extracts obtained at bench- and pilot-scales. Ind. Crop. Prod..

[25] Parada M.S., Fernández K. (2017). Modelling the hydrophilic extraction of the bark of Eucalyptus nitens and Eucalyptus globulus: Adsorption isotherm and thermodynamic studies. Ind. Crop. Prod..

[26] Astuya-Villalón A., Ziehe J., Rivera A., Ortiz S., Ulloa V., Roeckel M., Aspé E., Fernández K. (2016). Antioxidant and anti-inflammatory activities of *Pinus radiata* bark extract in salmonid cell lines. Aquac. Res..

[27] Jerez M., Selga A., Sineiro J., Torres J.L., Núñez M.J. (2005). A comparison between bark extracts from Pinus pinaster and *Pinus radiata*: Antioxidant activity and procyanidin composition. Food Chem..

[28] Chen G., Qiao C., Wang Y., Yao J. (2014). Synthesis of Biocompatible Gelatin-functionalised Graphene Nanosheets For Drug Delivery Applications. Aust. J. Chem..

[29] Ku C.S., Mun S.P. (2006). Characterization of proanthocyanidin in hot water extract isolated from *Pinus radiata* bark. Wood Sci. Technol..

[30] Ignatova M.G., Manolova N.E., Rashkov I., Markova N.D., Toshkova R.A., Georgieva A., Nikolova E.B. (2016). Poly(3-hydroxybutyrate)/caffeic acid electrospun fibrous materials coated with polyelectrolyte complex and their antibacterial activity and in vitro antitumor effect against HeLa cells. Mater. Sci. Eng. C.

[31] Faidi A., Lassoued M.A., Becheikh M.E.H., Touati M., Stumbé J.-F., Farhat F. (2019). Application of sodium alginate extracted from a Tunisian brown algae Padina pavonica for essential oil encapsulation: Microspheres preparation, characterization and in vitro release study. Int. J. Biol. Macromol..

[32] Khan A. (2018). Electrospinning of Crude Plant Extracts for Antibacterial and Wound Healing Applications: A Review. SM J. Biomed. Eng..

[33] Zhang W., Ronca S., Mele E. (2017). Electrospun Nanofibres Containing Antimicrobial Plant Extracts. Nanomaterials.

[34] Liu C., Wong H.M., Yeung K.W.K., Tjong S.C. (2016). Novel Electrospun Polylactic Acid Nanocomposite Fiber Mats with Hybrid Graphene Oxide and Nanohydroxyapatite Reinforcements Having Enhanced Biocompatibility. Polymers.

[35] Goudarzi Z.M., Behzad T., Ghasemi-Mobarakeh L., Kharaziha M., Enayati M.S. (2019). Structural and mechanical properties of fibrous poly (caprolactone)/gelatin nanocomposite incorporated with cellulose nanofibers. Polym. Bull..

[36] Jiang Z., Zhao L., He F., Tan H., Li Y., Tang Y., Duan X., Li Y. (2020). Palmatine-loaded electrospun poly(ε-caprolactone)/gelatin nanofibrous scaffolds accelerate wound healing and inhibit hypertrophic scar formation in a rabbit ear model. J. Biomater. Appl..

[37] Adeli-Sardou M., Yaghoobi M.M., Torkzadeh-Mahani M., Dodel M. (2018). Controlled release of lawsone from polycaprolactone/gelatin electrospun nano fibers for skin tissue regeneration. Int. J. Biol. Macromol..

[38] Salehi M., Shahporzadeh K., Ehterami A., Yeganehfard H., Ziaei H., Azizi M.M., Farzamfar S., Tahersoltani A., Goodarzi A., Ai J. (2020). Electrospun Poly(ε-caprolactone)/Gelatin Nanofibrous Mat Containing Selenium as a Potential Wound Dressing Material: In Vitro and In Vivo Study. Fibers Polym..

[39] Salehi M., Niyakan M., Ehterami A., Haghi-Daredeh S., Nazarnezhad S., Abbaszadeh-Goudarzi G., Vaez A., Hashemi S.F., Rezaei N., Mousavi S.R. (2019). Porous electrospun poly(ε-caprolactone)/gelatin nanofibrous mat containing cinnamon for wound healing application: In vitro and in vivo study. Biomed. Eng. Lett..

[40] Gomes S., Rodrigues G., Martins G., Roberto M., Mafra M., Henriques C., Silva J.C. (2015). In vitro and in vivo evaluation of electrospun nanofibers of PCL, chitosan and gelatin: A comparative study. Mater. Sci. Eng. C.

[41] Li N., Taylor L.S., Mauer L.J. (2011). Degradation Kinetics of Catechins in Green Tea Powder: Effects of Temperature and Relative Humidity. J. Agric. Food Chem..

[42] Li N., Taylor L.S., Ferruzzi M.G., Mauer L.J. (2012). Kinetic Study of Catechin Stability: Effects of pH, Concentration, and Temperature. J. Agric. Food Chem..

[43] Lin S., Chen M., Jiang H., Fan L., Sun B., Yu F., Yang X., Lou X., He C., Wang H. (2016). Green electrospun grape seed extract-loaded silk fibroin nanofibrous mats with excellent cytocompatibility and antioxidant effect. Colloids Surfaces B Biointerfaces.

[44] Díaz-Gómez R., Toledo-Araya H., López-Solís R., Obreque-Slier E. (2014). Combined effect of gallic acid and catechin against Escherichia coli. LWT.

[45] Ahamad S.T., Lakshmi T., RajeshKumar S., Roy A., Gurunadhan D., Geetha R. (2019). Antibacterial Activity of Taxifolin Isolated from Acacia Catechu Leaf Extract-An Invitro Study. Indian J. Public Health Res. Dev..

[46] Teixeira B., Marques A., Ramos C., Neng N.R., Nogueira J.M., Saraiva J.A., Nunes M.L. (2012). Chemical composition and antibacterial and antioxidant properties of commercial essential oils. Ind. Crop. Prod..

[47] Mirkarimi M., Amin-Marashi S.M., Bargrizan M., Abtahi A., Imani Fooladi A.A. (2012). The Antimicrobial Activity of Grape Seed Extract against Two Important Oral Pathogens. Zahedan J. Res. Med. Sci..

[48] Perumalla A.V.S., Hettiarachchy N.S. (2011). Green tea and grape seed extracts — Potential applications in food safety and quality. Food Res. Int..

[49] Rotava R., Zanella I., Silva L.P.D., Manfron M.P., Ceron C.S., Alves S.H., Karkow A.K., Santos J.P.A. (2009). Antibacterial, antioxidant and tanning activity of grape by-product/Atividade antibacteriana, antioxidante e tanante de subprodutos da uva. Cienc. Rural..

[50] Tesaki S., Tanabe S., Moriyama M., Fukushi E., Kawabata J., Watanabe M. (1999). Isolation and Identification of an Antibacterial Compound from Grape and Its Application to Foods. J. Agric. Chem. Soc. Jpn..

[51] Gazzarri M., Bartoli C., Mota C., Puppi D., Dinucci D., Volpi S., Chiellini F. (2013). Fibrous star poly(ε-caprolactone) melt-electrospun scaffolds for wound healing applications. J. Bioact. Compat. Polym..

[52] Schurer N., Kohne A., Schliep V., Barlag K., Goerz G. (1993). Lipid composition and synthesis of HaCaT cells, an immortalized human keratinocyte line, in comparison with normal human adult keratinocytes. Exp. Dermatol..

[53] Schoop V.M., Fusenig N.E., Mirancea N. (1999). Epidermal Organization and Differentiation of HaCaT Keratinocytes in Organotypic Coculture with Human Dermal Fibroblasts. J. Investig. Dermatol..

[54] Jahanshahi M., Hamdi D., Godau B., Samiei E., Sanchez-Lafuente C.L., Neale K.J., Hadisi Z., Dabiri S.M.H., Pagan E., Christie B.R. (2020). An Engineered Infected Epidermis Model for In Vitro Study of the Skin’s Pro-Inflammatory Response. Micromachines.

[55] Ku C.S., Mun S.P. (2011). Effect of Pinus radiate bark extracts with different molecular weight distributions on cell growth of NIH/3T3 fibroblasts and dendrite retraction of B16 melanoma cells. J. Wood Sci..

[56] Tsuruya M., Niwano Y., Nakamura K., Kanno T., Nakashima T., Egusa H., Sasaki K. (2014). Acceleration of Proliferative Response of Mouse Fibroblasts by Short-Time Pretreatment with Polyphenols. Appl. Biochem. Biotechnol..

[57] Unalan I., Boccaccini A.R. (2021). Essential oils in biomedical applications: Recent progress and future opportunities. Curr. Opin. Biomed. Eng..

