# Supplementary material for: Fabrication of Biocompatible Electrospun Poly(ε-caprolactone)/Gelatin Nanofibers Loaded with Pinus radiata Bark Extracts for Wound Healing Applications
Source: Polymers (Basel). 2022 Jun 9;14(12):2331. doi: 10.3390/polym14122331 (PMC9228265; doi:10.3390/polym14122331)
Supplement: Supplementary file 1 [file polymers-14-02331-s001.zip › polymers-1743062-supplementary.pdf]

Supplementary

Fabrication of Biocompatible Electrospun Poly (ε-caprolactone)/Gelatin Nanofibers Loaded with P*inus Radiata* Bark Extracts for Wound Healing Applications

Jessica Borges-Vilches [1], Irem Unalan [2], Katherina Fernández [1] and Aldo R. Boccaccini [2,*]

[1] Laboratory of Biomaterials, Department of Chemical Engineering, Faculty of Engineering, Universidad de Concepción, Concepción 4030000, Chile; jessborges@udec.cl (J.B.-V.); kfernandeze@udec.cl (K.F.)
[2] Institute of Biomaterials, Department of Materials Science and Engineering, Friedrich-Alexander-University Erlangen-Nuremberg, Cauerstraße 6, 91058 Erlangen, Germany; irem.unalan@fau.de
* Correspondence: aldo.boccaccini@ww.uni-erlangen.de

Figures and Tables

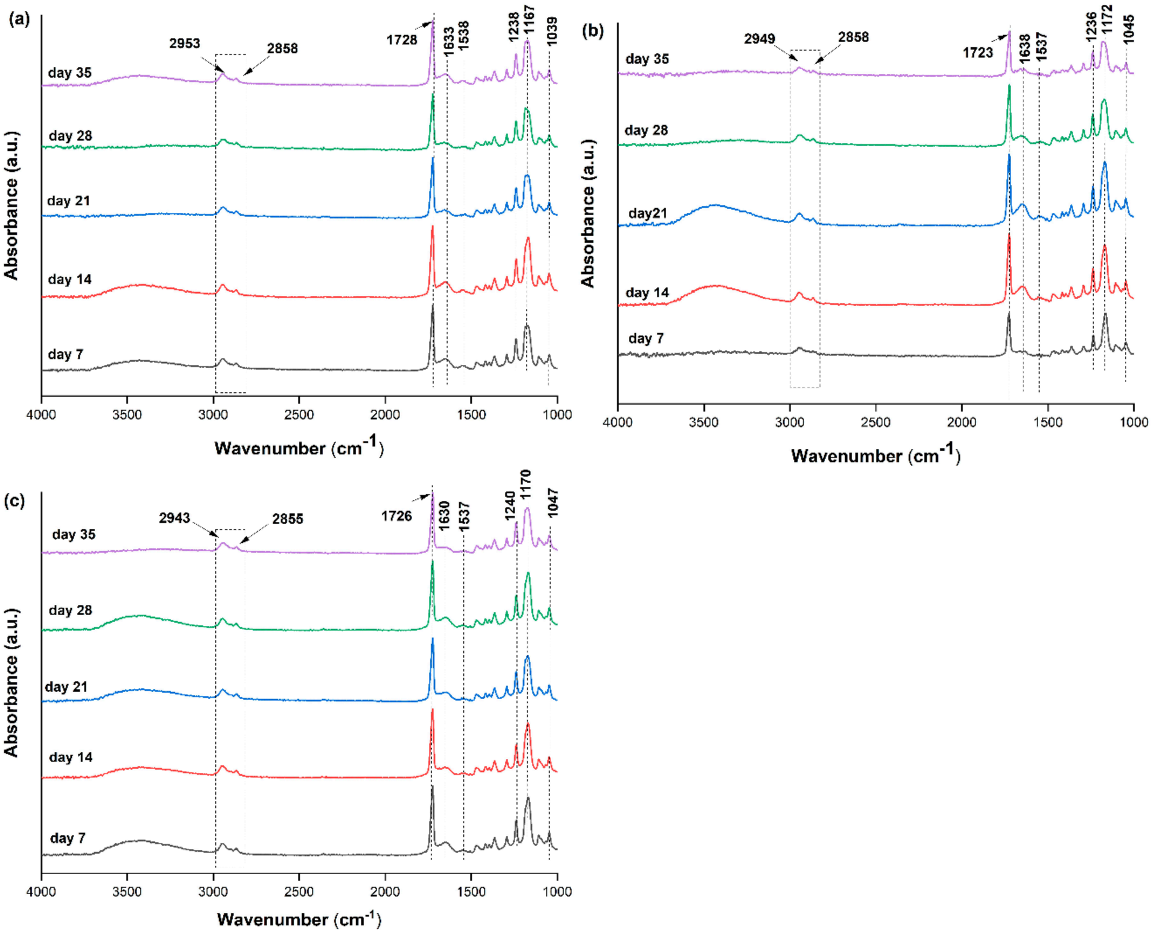

Figure S1. FTIR spectra obtained during the *in vitro* degradation assay of (a) PCL/GEL, (b) PCL/GEL/0.18%PE, and (c) PCL/GEL/0.36%PE electrospun nanofiber mats.

Table S1. Phenol composition and content of the *Pinus Radiata* bark extract.

Compounds	Content (mg per gram of extract)
(-)-Catechin	13.8
Taxifolin	13.9
p-Hydroxybenzoic acid	7.6
Homovanillic acid [a]	6.7

Quercetin	4.3
Proanthocyanidin B-2	3.3
(+)-Epicatechin	2.8
Dihydroxybenzoic acid [b]	2.7
Dihydroxybenzoic acid [b]	1.8
Syringic acid [a]	1.1
3,4-dihydroxyphenyl acetic acid	0.9
Dihydroxybenzoic acid [b]	0.2
Epigallocatechin	n.d

n.d.: not detected, a: tentatively identified compounds, b: not recognized isomers.

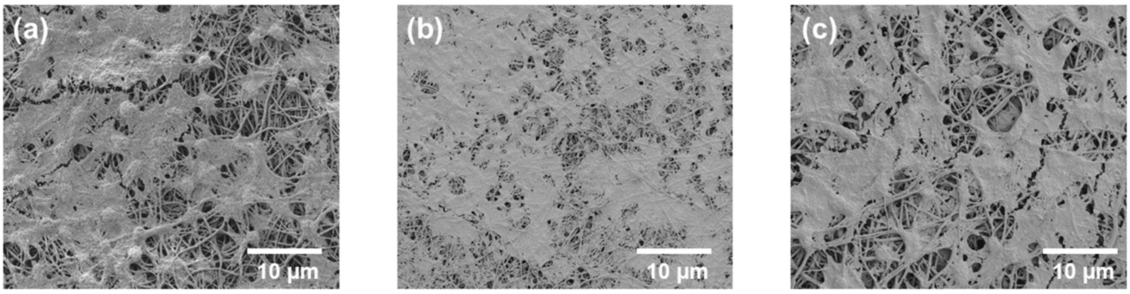

Figure S2. SEM micrographs of HaCaT cells cultured on (a) PCL/GEL, (b) PCL/GEL/0.18%PE, and (c) PCL/GEL/0.36%PE electrospun nanofiber mats. The images were taken after incubation and staining studies.

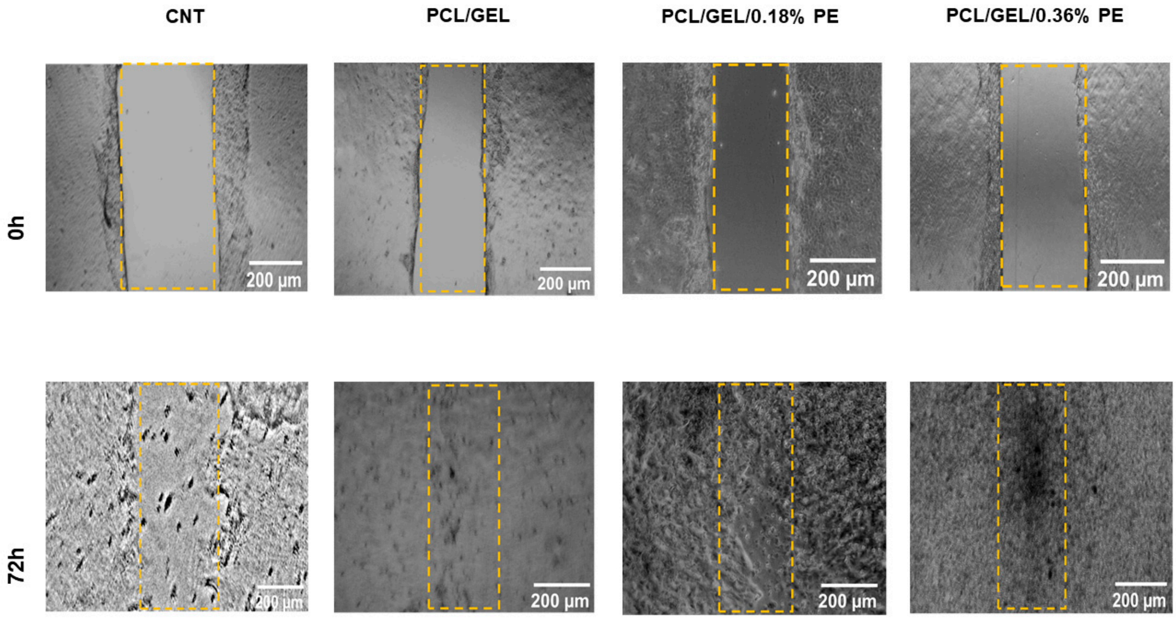

Figure S3. Micrographs of the cell migration into a scratch area over a 72h period in CNT, PCL/GEL, PCL/GEL/0.18%PE, and PCL/GEL/0.36%PE electrospun nanofiber mats, respectively.
